# Polymeric microarray patches for transdermal delivery of amodiaquine and artesunate: A novel strategy against *Plasmodium falciparum*

**DOI:** 10.1016/j.mtbio.2025.102500

**Published:** 2025-10-31

**Authors:** Qonita Kurnia Anjani, Fabiana Volpe-Zanuto, Andang Miatmoko, Natalia Moreno-Castellanos, Janaina Tenorio Novais, Xiomara A. Gaitán, Berlian Sarasitha Hariawan, Devy Maulidya Cahyani, Rifda Tarimi Octavia, Ahmad Shahrul Mubarok, Wiwit Ananda Wahyu Setyaningsih, Callum Johnston, Daniel Y. Bargieri, Ryan F. Donnelly

**Affiliations:** a School of Pharmacy, Queen's University Belfast, Medical Biology Centre, 97 Lisburn Road, Belfast, BT9 7BL, UK; b School of Biomedical Sciences, Ulster University, Mass Spectrometry Centre, Cromore Road, Coleraine, BT52 1SA, UK; c Department of Pharmaceutical Science, Faculty of Pharmacy, Universitas Airlangga, Campus C UNAIR Mulyorejo, Surabaya, 60115, Indonesia; d Stem Cell Research and Development Center, Universitas Airlangga, Institute of Tropical Disease Building, Campus C UNAIR Mulyorejo, Surabaya, 60115, Indonesia; e Basic Science Department, Faculty of Health, Universidad Industrial de Santander, Bucaramanga, 680001, Colombia; f Department of Parasitology, Institute of Biomedical Sciences, University of São Paulo, São Paulo, Brazil; g Department of Chemistry, Faculty of Science and Data Analytics, Institut Teknologi Sepuluh Nopember, Sukolilo, Surabaya, 60111, Indonesia; h Department of Nanotechnology Engineering, Faculty of Advanced Technology and Multidiscipline, Universitas Airlangga, Campus C UNAIR Mulyorejo, Surabaya, 60115, Indonesia; i The Wellcome-Wolfson Institute for Experimental Medicine, Queen's University Belfast, 97 Lisburn Road, Belfast, BT9 7BL, UK; j Department of Anatomy, Faculty of Medicine, Public Health, and Nursing, Universitas Gadjah Mada, Sleman, D.I. Yogyakarta, 55281, Indonesia

**Keywords:** Dissolving microarray patches, Transdermal drug delivery, Amodiaquine, Artesunate, Malaria treatment

## Abstract

Malaria remains a significant global health burden, particularly in low- and middle-income countries. Artemisinin-based combination therapies (ACTs) are the current gold standard for treating *Plasmodium falciparum* infections, combining a fast-acting artemisinin derivative with a longer-acting partner drug. However, these oral regimens require multiple doses over several days, which can reduce adherence, compromise parasite clearance, and contribute to the emergence of drug resistance. To overcome these limitations, we developed, for the first time, dissolving microarray patches (MAPs) for the transdermal delivery of amodiaquine and artesunate. Given the different Biopharmaceutics Classification System (BCS) properties of these drugs, amodiaquine (BCS Class III) and artesunate (BCS Class II), Tween® 80 was incorporated to enhance solubility and skin permeability. *In vitro* release studies demonstrated efficient delivery, with up to 40 % amodiaquine and 90 % artesunate deposited and permeated into the skin. Pharmacokinetic analysis revealed that MAP delivery extended the half-life of artesunate by 8.5-fold (54.05 *vs.* 6.37 h) and increased its C_max_ by 21 % (1796.02 ± 154.50 *vs.* 1421.93 ± 209.61 ng/mL), while amodiaquine's half-life was prolonged by ∼1.4-fold (57.72 ± 19.16 *vs.* 40.75 ± 7.44 h). In a *Plasmodium berghei*-infected murine model, the combined MAP treatment reduced parasitaemia by 99.5 % within seven days, showing comparable efficacy to oral administration. These findings demonstrate that dissolving MAPs offer a minimally invasive, needle-free strategy for ACT delivery, with potential to enhance treatment adherence, reduce gastrointestinal side effects, and combat drug resistance, particularly in resource-limited malaria-endemic settings.

## Highlights

• Dissolving MAPs were developed for transdermal delivery of artesunate and amodiaquine.
• Tween® 80 improved solubility and skin permeation of both antimalarial drugs.
• MAPs extended the half-life of artesunate by over 8-fold compared to oral dosing.
• Combined MAPs reduced parasitaemia by 99.5 % in a murine malaria model.
• This needle-free platform may improve ACT adherence in malaria-endemic settings.

## Graphical abstract

**Figure:**
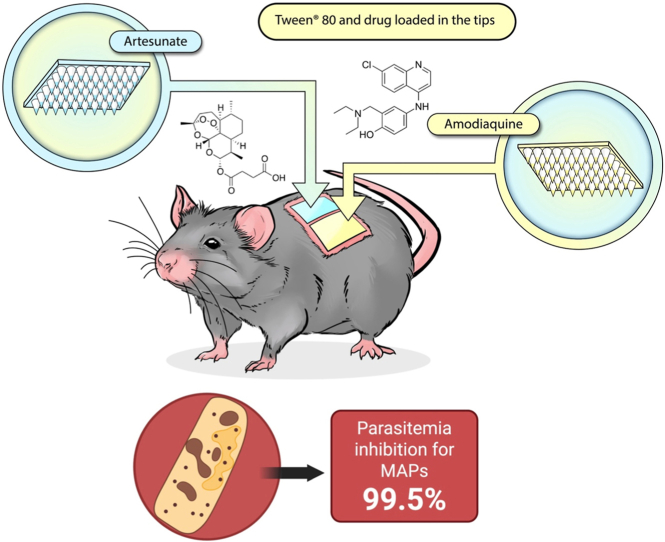

## 1 Introduction

Malaria remains one of the most devastating infectious diseases, disproportionately affecting low- and middle-income countries, particularly in sub-Saharan Africa and Southeast Asia [[1], [2], [3]]. The disease is caused by Plasmodium parasites, with *Plasmodium falciparum* being the most lethal species, responsible for the highest mortality rates [4]. According to the World Health Organization (WHO), malaria accounted for an estimated 263 million cases and over 597,000 deaths in 2023, with children under five and pregnant women being the most vulnerable populations [5]. Due to climate change, the number of people at risk is projected to increase by 1.6 million by 2030 and 1.8 million by 2050 [6]. Despite advancements in malaria control strategies, including vector management and the development of antimalarial drugs, the disease continues to pose a significant public health burden.

To combat malaria, artemisinin-based combination therapies (ACTs) remain the gold standard for treating uncomplicated *P. falciparum* infections [7,8]. These therapies pair a short-acting artemisinin derivative, such as artesunate, with a longer-acting partner drug, such as amodiaquine, to achieve rapid parasite clearance and prevent relapse [9,10]. This specific combination is widely endorsed by the WHO and is especially important in sub-Saharan Africa, where it is used as a first-line treatment, particularly in paediatric populations [11]. However, the standard oral administration of ACTs presents several limitations. Oral regimens require multiple daily doses over three to seven days, often resulting in poor adherence, incomplete parasite elimination, and an increased risk of resistance [12]. Additionally, gastrointestinal side effects such as nausea and vomiting frequently lead to treatment discontinuation [13]. Artesunate is rapidly hydrolysed to its active metabolite, dihydroartemisinin (DHA), which has a short half-life, necessitating frequent dosing to maintain effective plasma concentrations [14]. Amodiaquine, while providing longer-term protection, undergoes extensive first-pass metabolism, contributing to variability in therapeutic levels and reduced bioavailability [15]. These pharmacokinetic constraints, rapid clearance and complication in the dose required for artesunate and hepatic metabolism for amodiaquine, highlight the need for an alternative delivery strategy.

To address these challenges, transdermal drug delivery *via* microarray patch (MAP)-based platforms has gained increasing interest as a minimally-invasive, self-administrable alternative to conventional routes. MAPs create micropores in the *stratum corneum*, facilitating drug permeation into the dermal microcirculation, thereby bypassing the GI tract and first-pass metabolism [16]. Among the different microneedle technologies, dissolving MAPs offer several advantages for malaria treatment, including minimal invasiveness, eliminating the pain associated with injections and improving patient compliance; the absence of biohazardous waste, unlike hypodermic needles, which require proper disposal; self-administration potential, enabling use in remote malaria-endemic areas without healthcare supervision; and sustained drug release, ensuring controlled and prolonged drug delivery, thereby reducing dosing frequency [16,17].

Our previous studies have demonstrated the feasibility of dissolving MAPs for antimalarial drug delivery [[18], [19], [20]]. MAP-based delivery of artemisinin derivatives has shown improved bioavailability, stability, and a more rapid therapeutic onset compared to oral formulations [19]. However, the biopharmaceutical classification system (BCS) properties of amodiaquine and artesunate present unique formulation challenges that must be addressed for effective transdermal drug delivery. Amodiaquine is classified as BCS Class III, characterised by high solubility but poor permeability, which limits its passive skin permeation, necessitating an enhanced transdermal delivery mechanism such as MAPs to facilitate absorption [21]. Conversely, artesunate belongs to BCS Class II, exhibiting low solubility but high permeability, meaning that solubility enhancement strategies are required to improve its dispersion within the microneedle matrix [22].

In this study, we report for the first time, the development of dissolving MAPs for the transdermal delivery of amodiaquine and artesunate, two essential components of ACTs. This novel formulation strategy addresses key pharmacokinetic and adherence challenges associated with conventional oral ACTs. The MAPs are designed to deliver fast-acting artesunate for rapid parasite clearance, alongside sustained-release amodiaquine for prolonged therapeutic protection. Importantly, we incorporated Tween® 80 to overcome the solubility and permeability limitations posed by the distinct BCS properties of the two drugs. This MAP platform offers a minimally invasive, needle-free, and potentially self-administered alternative to oral and injectable antimalarials, particularly suited for paediatric and resource-limited settings. The outcomes of this study provide the first pharmacokinetic and efficacy evidence for this MAP strategy, laying the groundwork for clinical translation and large-scale deployment in malaria-endemic regions.

## 2 Materials and methods

### 2.1 Materials

Amodiaquine dihydrochloride and artesunate, both with a purity of 97 %, were obtained from Fluorochem (Hadfield, UK). Poly(vinyl alcohol) (PVA; 9-10 kDa), poly(vinyl pyrrolidone) K90 (PVP; 360 kDa), and sodium carboxymethyl cellulose were supplied by Sigma-Aldrich (Dorset, UK). PVP K17 or Kollidon® 17PF (7–11 kDa) was generously provided by BASF (Ludwigshafen, Germany). Cell culture reagents including Dulbecco's Modified Eagle Medium (DMEM), 3-(4,5-dimethylthiazol-2-yl)-2,5-diphenyltetrazolium bromide, fetal bovine serum (FBS), penicillin-streptomycin, and Triton X-100 were also sourced from Sigma-Aldrich (St. Louis, MO, USA). Calcein AM and ethidium homodimer-1 dyes were purchased from Molecular Probes (Eugene, OR, USA). The Quant-iT™ PicoGreen™ dsDNA assay kit and reagent were obtained from Thermo Fisher Scientific (Waltham, MA, USA). Ultrapure water used throughout the study was generated using a PURELAB DV 25 system from Elga (Veolia Water Systems, Dublin, Ireland). All other chemicals and reagents were acquired from Sigma-Aldrich (Dorset, UK). Neonatal porcine skin was harvested from stillborn piglets within 24 h of delivery and stored at −20 °C until required.

### 2.2 Saturation solubility

An excess quantity of either amodiaquine or artesunate was introduced into separate Eppendorf tubes containing 1 mL of deionised water, phosphate-buffered saline (PBS, pH 7.4), or a 0.5 % w/v Tween® 80 solution. The contents of each tube were vortexed (Fisons Scientific Equipment, Loughborough, Leicestershire, UK) for 1 min at 3500 rpm to facilitate thorough mixing. Samples were subsequently incubated at 37 °C with shaking at 1500 rpm for 24 h using a shaker incubator (Jeio Tech ISF-7100, Medline Scientific, Chalgrove, UK). Following incubation, the suspensions were centrifuged at 14,800 rpm for 15 min to precipitate undissolved material. The supernatants were then filtered through a 0.45 μm nylon membrane filter. Where needed, the filtrates were diluted with PBS prior to high-performance liquid chromatography (HPLC) analysis.

### 2.3 Fabrication of dissolving MAPs

**Fig. 1:**
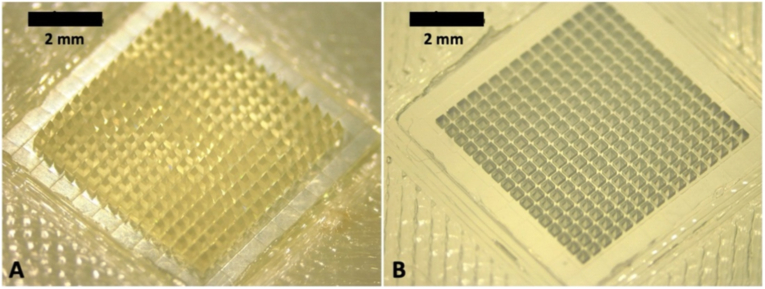
Representative images of (A) the 3D-printed master moulds and the corresponding (B) poly(dimethylsiloxane) (PDMS) moulds used in this study for the fabrication of dissolving MAPs.

Dissolving MAPs were formulated using a double-casting technique, as previously published [[23], [24], [25], [26], [27]], with a slight modification. The first casting step, designed to form the needle tips, involved incorporating amodiaquine or artesunate into a polymer blend consisting of 20 % w/w PVA (9–10 kDa), 20 % w/w PVP K17 (7-11 kDa), and either deionised water or a Tween® 80 surfactant solution, as outlined in Table 1. The second layer, which served as the baseplate, was composed of an aqueous mixture containing 30 % w/w PVP K90 (360 kDa) and 1.5 % w/w glycerol. For the tip layer, 50 mg of the drug–polymer formulation was dispensed into poly(dimethylsiloxane) (PDMS) moulds featuring a 16 × 16 needle array with pyramidal-cuboidal geometry (850 μm height, 300 μm base width, 300 μm spacing; total area: 0.36 cm^2^). These PDMS moulds were fabricated using 3D-printed engineered male resin moulds with the same dimensions, as illustrated in Fig. 1A, and the resulting PDMS mould is shown in Fig. 1B. The filled moulds (with the first layer formulation) were centrifuged (Centrifuge 5804R, Eppendorf®, Eppendorf SE, Hamburg, Germany) to ensure complete cavity filling, and any excess formulation was carefully removed using a spatula. Elastomer rings (23 mm outer diameter, 18 mm inner diameter, 3 mm thickness) were attached to the moulds using a 40 % w/w PVA (9–10 kDa) adhesive to secure the structure, and the assemblies were left to dry at ambient temperature overnight. The elastomer ring was used to contain the baseplate formulation during centrifugation, allowing uniform filling of the mould and complete MAP formation. For baseplate layer, 500 μL of the PVP K90-glycerol solution was added to each mould and centrifuged at 3500 rpm for 10 min. The moulds were again air-dried at room temperature for 24 h. Any sidewalls formed during the casting process were trimmed with scissors, and a final drying step was carried out at 37 °C overnight.

**Table 1:** Composition of the first-layer needle formulation in dissolving MAPs containing amodiaquine or artesunate.

Components (mg in 100 mg of aqueous mixture)	F1 (pure drug)	F2 (pure drug)	F1	F2	F3	F4
Drug (amodiaquine or artesunate)	20 mg	40 mg	20 mg	40 mg	20 mg	40 mg
Aqueous polymer blend (PVA and PVP K17 (20 % w/w)	40 mg	40 mg	40 mg	40 mg	20 mg	20 mg
Deionised water	40 mg	20 mg	–	–	–	–
Tween® 80 solution (0.5 % w/v)	–	–	40 mg	20 mg	60 mg	40 mg

### 2.4 Drug loading quantification

The drug content in the MAPs was determined by dissolving the formulation in deionised water and making up to 4 mL. To facilitate dissolution, the MAPs underwent a 30-min sonication cycle using a bath sonicator (Ultrawave Ltd., Cardiff, UK), Subsequently, 4 mL of methanol was added to fully solubilise the amodiaquine or artesunate, followed by an additional 30 min of sonication. The resulting mixture was then centrifuged (Centrifuge 5425R, eppendorf®, Eppendorf SE, Hamburg, Germany) at 14,500 rpm for 15 min. The supernatant was filtered and collected and analysed using HPLC to quantify the drug content.

### 2.5 Morphology and mechanical properties evaluation

The morphology of the dissolving MAPs was characterised using an light microscope (Leica EZ4 D, Leica Microsystems, Milton Keynes, UK) and a scanning electron microscope (SEM) (SNE Alpha SEM, Seiko Instruments and Electronics Ltd., New Taipei City, Taiwan). Mechanical strengths were assessed by measuring the percentage reduction in needle height following compression. This evaluation was performed using a TA-TX2 Texture Analyser (Stable Microsystems, Haslemere, UK), following established procedures. For this test, the MAPs were positioned on the metallic base of the Texture Analyser with the needle side facing downward, against a flat metal block. The device operated in compression mode with a pre-test, test, and post-test speed of 1 mm/s. A compressive force of 32 N was applied for 30 s, and the needle height reduction was calculated as a percentage of the initial needle height. This downward-facing configuration was chosen to simulate real-life MAP application, in which the force is applied onto the baseplate (*via* thumb pressure) to drive the needles into the skin. As such, this setup allows for a practical assessment of mechanical performance under clinically relevant loading conditions.

### 2.6 Insertion assessment

The insertion capability of the dissolving MAPs was evaluated using both an 8-layer Parafilm® M stack and *ex vivo* neonatal porcine skin. An EX-101 optical coherence tomography (OCT) microscope (Michelson Diagnostics Ltd., Kent, UK) was used for visualisation, following protocols adapted from previously published methods [28,29]. Before testing, excised porcine skin samples were pre-equilibrated in PBS. The MAPs were applied to either the Parafilm® M or the *ex vivo* porcine skin using a TA-TX2 Texture Analyser, which exerted a compressive force of 32 N for 30 s to replicate thumb pressure during manual application. OCT was used to image the insertion sites, and penetration depth was quantified using ImageJ® software (National Institutes of Health, Bethesda, MD, USA). In the case of Parafilm® stacks, the layers were separated after application and inspected under a light microscope to count and determine the number of puncture holes formed.

### 2.7 *In situ* skin dissolution study

The *in situ* dissolution behaviour of the dissolving MAPs was studied using excised full-thickness neonatal porcine skin, with an approximate thickness of 1.5 mm. Prior to application, the skin was immersed in PBS (pH 7.4) for 30 min to allow equilibration. Each MAP was manually inserted into the prepared skin using firm thumb pressure for 30 s. To maintain consistent contact and prevent movement during incubation, a stainless-steel cylindrical weight (15 g; 1.4 cm diameter, 1.0 cm height) was positioned directly over the MAP. The samples were incubated at 37 °C for durations of 1, 2, and 3 h. At each time point, MAPs were gently removed and observed under a light microscope to evaluate the extent of needle dissolution.

### 2.8 Dermatokinetic study

Dermatokinetic assessments were performed using full-thickness neonatal porcine skin to investigate the intradermal distribution of amodiaquine and artesunate following application *via* dissolving MAPs. The study was carried out using 12 mL vertical Franz diffusion cells (PermeGear, Hellertown, PA, USA). Skin samples were secured to the donor chamber with cyanoacrylate adhesive (Stick it® super glue, PLDZ Pattison House, Dublin, Ireland). Degassed phosphate-buffered saline (PBS) was used as the receptor fluid, maintained at 37 ± 1 °C and stirred continuously at 600 rpm. A MAP containing either drug was applied manually with firm thumb pressure for 30 s. A stainless-steel cylindrical weight was placed on the top of MAP baseplate and then the diffusion cell was assembled. A drug-loaded film of identical concentration, but without microneedles, served as the control.

To prevent solvent evaporation, Parafilm® was used to seal the donor compartment and sampling port. At predetermined time points, cells were disassembled and 200 μL samples of receptor fluid were withdrawn, filtered, and analysed by HPLC. Following drug permeation, skin samples were briefly heated at 60 °C for 5 min to facilitate epidermis-dermis separation [25,26,30,31]. The epidermal tissue was homogenised in 2 mL of methanol using a mixer (ThermoMixer™F2.0, Eppendorf, Hamburg, Germany) for 30 min, whereas dermal tissue was processed in 0.5 mL of deionised water using a Tissue Lyser LT (Qiagen Ltd., Manchester, UK) at 50 Hz for 15 min. An additional 1 mL of methanol was then added to the dermis samples, followed by a second round of homogenisation. The resulting homogenates from both layers were centrifuged at 14,000 rpm for 15 min. Supernatants were filtered through a 0.45 μm PTFE membrane (25 mm diameter; Agilent Technologies UK Ltd., Stockport, UK) prior to HPLC analysis.

### 2.9 Biocompatibility study

Biocompatibility of the formulated MAPs, including blank, amodiaquine-loaded, and artesunate-loaded MAPs, was evaluated using adult human dermal fibroblasts (HDFa). Assessments included measurements of cell viability, cytotoxicity, and proliferation. The MTT assay was employed to quantify cell viability and toxicity. HDFa cells were seeded at a density of 5 × 10^3^ cells per well and cultured for 72 h in Dulbecco's Modified Eagle Medium (DMEM) supplemented with 10 % fetal bovine serum (FBS) and 1 % penicillin-streptomycin. Following the incubation period, 5 mg/mL MTT solution was added, and the cells were incubated at 37 °C in a humidified 5 % CO_2_ environment for an additional 5 h. After treatment, the MAPs were rinsed with PBS, and the resulting formazan crystals were solubilised in DMSO. Absorbance was recorded at 570 nm using a Multiskan GO spectrophotometer (Thermo Fisher Scientific, Waltham, MA, USA). Untreated cells were used as a positive control, while treatment with 1 % Triton X-100 served as a negative control. Cell proliferation was evaluated by determining DNA content using the Quant-iT™ PicoGreen™ dsDNA assay. After exposure, samples were rinsed three times with PBS (pH 7.4) and lysed in 1 mL of lysis buffer containing 10 mM Tris (pH 8.0), 1 mM EDTA, and 0.2 % Triton X-100. To ensure efficient DNA extraction, the samples were vortexed for 10 s every 5 min over a 30-min period while maintained on ice. Homogenisation was performed for 10–15 min, followed by mixing with 100 μL of PicoGreen fluorescent dye. Fluorescence was measured at an excitation wavelength of 480 nm and emission at 520 nm. DNA concentrations were determined using a standard curve prepared with lambda DNA. All tests were conducted in triplicate.

### 2.10 *In vivo* pharmacokinetic study

The pharmacokinetic study was carried out at the Stem Cell Research and Development Center, Universitas Airlangga, Surabaya, Indonesia, following the ethical guidelines set by the Animal Care and Use Committee (ACUC) of the Faculty of Veterinary Medicine, Airlangga University (approval number: 2.KEH.104.07.2024). Six female Sprague Dawley rats (four weeks old) had their back fur shaved using electric clippers. The following day, sedation was induced *via* intraperitoneal injection of ketamine (50 mg/kg) and xylazine (20 mg/kg). Once sedated, four dissolving MAPs containing amodiaquine or artesunate were applied to the back of each rat and secured with Microfoam™ surgical tape (3M, Bracknell, UK), followed by Kinesiology™ tape (Proworks, Stockton, UK), and left in place for 24 h. Each MAP contained approximately 5.5 mg of amodiaquine or 4.8 mg of artesunate. Accordingly, the total drug dose administered *via* MAPs (each rat received four patches) was 22 mg for amodiaquine and 19.2 mg for artesunate. For comparison, the oral administration group received a single dose of amodiaquine (10 mg/kg) and artesunate (4 mg/kg) by oral gavage following overnight fasting, based on previously published protocols [32,33]. Blood samples were collected from the tail vein at predetermined time points (0, 1, 2, 4, 6, 24, 48, and 72 h) using heparinised syringes. Plasma was separated from the whole blood and stored at −80 °C until further analysis.

### 2.11 Drug extraction from plasma

Following blood collection, samples were immediately centrifuged at 2000×*g* for 15 min at 4 °C using a temperature-controlled centrifuge (Sigma 2–16 K, Osterode am Harz, Germany). The plasma was carefully separated and stored at −80 °C until further analysis. For drug extraction, 50 μL of plasma was used for amodiaquine quantification, while 100 μL was allocated for artesunate analysis. To precipitate plasma proteins, 400 μL of ethyl acetate was added to the amodiaquine samples, and 180 μL of ethyl acetate was added to the artesunate samples. The mixtures were vortexed for 1 min to ensure complete extraction. Subsequently, the tubes were centrifuged at 3500 rpm for 10 min. The supernatants, 350 μL for amodiaquine and 150 μL for artesunate, were carefully transferred into clean Eppendorf tubes. The collected supernatants were placed in a fume hood for overnight to allow complete evaporation of the solvent. The dried residues were then reconstituted with 200 μL of an acetonitrile and deionised water solution (90:10 v/v) for amodiaquine, and 100 μL of the same mixture for artesunate. Finally, the prepared samples were analysed using high-performance liquid chromatography coupled with tandem mass spectrometry (HPLC-MS/MS; Agilent Technologies UK Ltd, Stockport, UK) for quantitative determination of drug concentrations.

### 2.12 High-performance liquid chromatography (HPLC) analysis

For the *in vitro* quantification of amodiaquine and artesunate, reversed-phase HPLC was conducted using an Agilent Technologies 1220 Infinity Compact LC system (Agilent Technologies UK Ltd, Stockport, UK) equipped with a UV detector. Chromatographic separation was achieved using an XSelect CSH C18 column (3.0 mm internal diameter, 150 mm length, 3.5 μm particle size, 130 Å pore size) from Waters (Dublin, Ireland), with a VanGuard® pre-column cartridge (3.9 mm internal diameter, 5 mm length) of matched chemistry used to protect the main column. The mobile phase consisted of 0.1 % v/v trifluoroacetic acid and acetonitrile in a 20:80 v/v ratio. The system was operated at a flow rate of 0.6 mL/min and a column temperature of 30 °C. Injection volumes were 10 μL for amodiaquine and 40 μL for artesunate, with analysis times of 5 and 7 min, respectively. Detection wavelengths were 210 nm for amodiaquine and 341 nm for artesunate.

For *in vivo* plasma analysis, samples were analysed using an Agilent 1200 Series HPLC system (Agilent Technologies UK Ltd, Stockport, UK) coupled to a Thermo Scientific TSQ Vantage triple quadrupole mass spectrometer. Mass spectrometry was performed in positive electrospray ionisation mode using multiple reaction monitoring (MRM) with the following parameters: spray voltage 3000 V, vaporiser temperature 200 °C, sheath gas 35 psi, auxiliary gas 10 psi, capillary temperature 270 °C, and S-Lens RF amplitude 129. Chromatographic separation was conducted on a ZORBAX Eclipse XBD-C18 column (1.8 μm, 4.6 × 50 mm; Agilent, Santa Clara, CA, USA). For artesunate, a 10-min isocratic method was employed using a mobile phase of 30 % v/v ammonium formate (5 μM) and 70 % v/v 0.1 % formic acid in acetonitrile, at a flow rate of 0.4 mL/min. The column temperature was 30 °C, injection volume 20 μL, and detection wavelength 210 nm. The ammonium adduct of artesunate (402 *m/z*) was monitored with transitions to 163.10 *m/z* (CE: 26 V) and 221.24 *m/z* (CE: 15 V). Dihydroartemisinin (DHA), its active metabolite, was monitored with a precursor ion at 284.35 *m/z* and transitions to 249.15 *m/z* (CE: 15 V) and 179.10 *m/z* (CE: 47 V). For amodiaquine, a 40-min gradient method was used with mobile phase A (0.1 % formic acid in water) and mobile phase B (0.1 % formic acid in acetonitrile). The gradient profile was: 95 % A/5 % B (0–5 min), 5 % A/95 % B (30–35 min), followed by re-equilibration (35.1–40 min). The flow rate was 0.4 mL/min, column temperature 20 °C, injection volume 15 μL, and detection wavelength 250 nm. Amodiaquine was detected using ion transitions from 356.115 *m/z* to 283.03 *m/z* with a collision energy of 23 V.

### 2.13 Histopathology

To evaluate the potential hepatotoxicity of the formulations, liver samples were collected *post-mortem* from the treated rats. The collected tissues were fixed in 4 % paraformaldehyde to preserve their structural integrity and subsequently embedded in paraffin blocks. Thin sections of 5 μm were prepared using a microtome for histological analysis. These sections were stained with haematoxylin and eosin (H&E) to enable visualisation of cellular and tissue architecture. Coverslips were mounted onto the slides using an optical-grade adhesive to protect the samples and facilitate imaging. Digital slide scanning was performed using the Aperio® AT2 digital pathology system (Leica Microsystems Ltd., Sheffield, UK). For each tissue type, 10–15 fields of view were systematically analysed to assess histological changes. Liver sections were evaluated for sinusoidal congestion, hepatocyte cytoplasmic vacuolisation, and parenchymal necrosis. The scoring system, ranging from 0 (no damage) to 4 (severe damage), was applied according to the method described by Suzuki *et al.* [34], as outlined in Table 2. This approach ensured a detailed and quantifiable assessment of potential histopathological changes related to MAP application.

**Table 2:** Suzuki scoring system for evaluating liver damage following hepatic ischemia/reperfusion injury [34].

Score	Congestion	Vacuolisation	Necrosis
0	None	None	None
1	Minimal	Minimal	Single necrosis
2	Mild	Mild	−30 %
3	Moderate	Moderate	−60 %
4	Severe	Severe	>60 %

### 2.14 *In vivo* antimalarial activity in *Plasmodium berghei*-infected mice

The formulation efficacy study was conducted at the animal facility of the Institute of Biomedical Sciences, University of São Paulo, Brazil. The ability of the MAPs to inhibit parasitaemia was assessed over a seven-day period using female BALB/c C57BL/6 mice (4 weeks old, 20 ± 3 g) infected with *Plasmodium berghei*. The study was approved by the Ethics Committee on the Use of Animals (CEUA) under registration numbers 1810220724 and 9215030119. The mice were housed in a pathogen-free environment in polypropylene cages and randomly assigned into three groups (n = 7 per group): a control group receiving blank MAPs (without drug), a group treated with amodiaquine and artesunate *via* oral administration, and a group treated with the same drugs delivered *via* MAP application. On day 0, mice assigned to the MAP group were shaved under anaesthesia, and all groups were infected *via* intraperitoneal injection with 1 × 10^5^ *P. berghei* ANKA HSP70-GFP-infected erythrocytes. Treatment commenced 24 h post-infection. MAPs were applied to the respective group and left in place for 24 h, in accordance with a modified Peter's 4-day suppressive test protocol [18,35]. Parasitaemia levels were assessed daily by flow cytometric analysis of whole blood samples (1 drop of blood mixed with 400 μL PBS), detecting 100,000 events per sample (FACSCalibur; 488 nm laser, 540 nm emission filter), until the conclusion of treatment. Parasitaemia inhibition was calculated as a percentage relative to the blank MAP (untreated control) group [36].

### 2.15 Statistical analysis

Statistical analysis was conducted using GraphPad Prism® version 10.4.2 (GraphPad Software, San Diego, CA, USA). Data are presented as means ± standard deviation (SD), unless otherwise specified. Differences among multiple groups were evaluated using one-way analysis of variance (ANOVA), while comparisons between two groups were analysed using Student's t-test. A *p*-value of less than 0.05 (*p* < 0.05) was regarded as statistically significant for all analyses.

## 3 Results and discussion

### 3.1 Saturation and solubility study

**Fig. 2:**
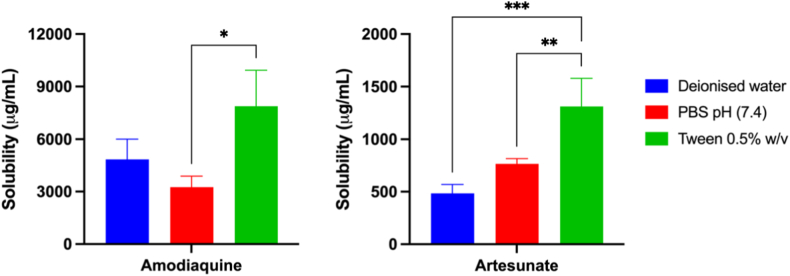
Saturation solubility of amodiaquine and artesunate in deionised water, PBS (pH 7.4), and Tween® 80 (0.5 % w/v) solution (means +SD, n = 4).

The solubility of amodiaquine and artesunate in various media, including deionised water, PBS (pH 7.4), and a 0.5 % w/v Tween® 80 solution, is illustrated in Fig. 2. No significant difference in solubility was observed between deionised water and PBS for both drugs (*p* > 0.05, both drugs). However, the highest solubility for both amodiaquine and artesunate was recorded in the 0.5 % w/v Tween® 80 solution, reaching approximately 7.9 ± 2.1 mg and 1.3 ± 0.3 mg, respectively, which was significantly higher than in PBS (*p* < 0.05, all cases). This finding is consistent with previous studies demonstrating that the inclusion of Tween® 80 in MAP formulations enhances solubility, improves drug performance, and increases delivery efficiency for both hydrophilic and hydrophobic drugs [31,[37], [38], [39]]. As a non-ionic surfactant, Tween® 80 has a lower potential for irritation compared to ionic surfactants and is generally considered safer [40,41]. It interacts with skin lipids by temporarily increasing the fluidity of biological membranes [42]. These results suggest that the incorporation of surfactants can improve the dispersion of amodiaquine and artesunate within formulations, leading to higher drug content and faster MAP tip dissolution. Consequently, enhancing solubility through surfactant incorporation could contribute to more effective transdermal drug delivery.

### 3.2 Fabrication of dissolving MAPs

**Fig. 3:**
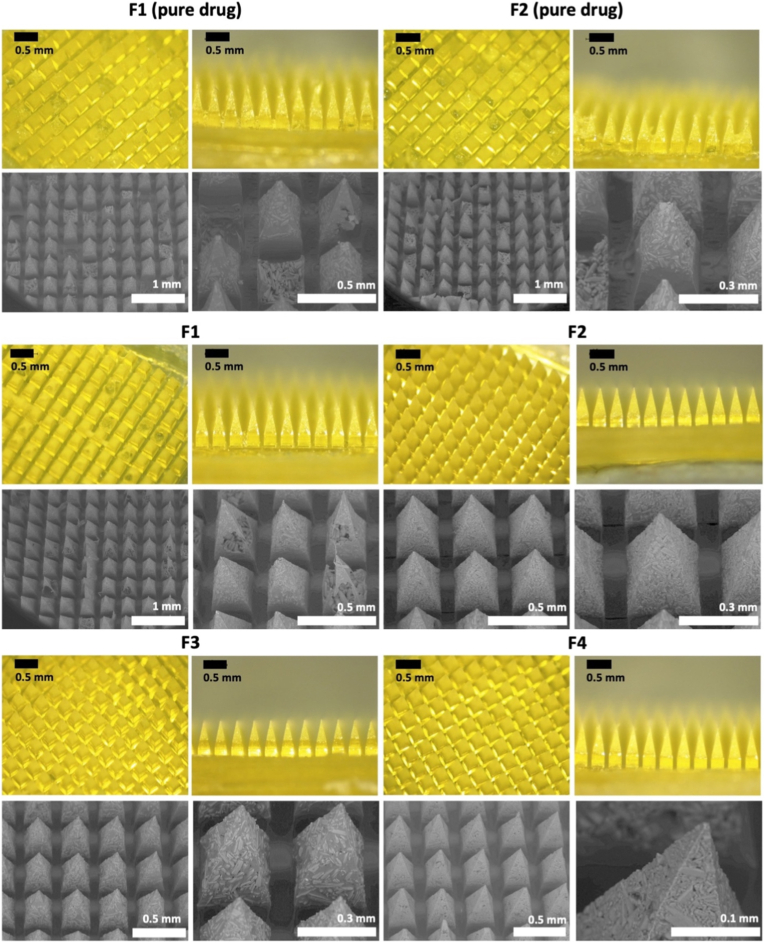
Optical characterisation of amodiaquine-loaded MAPs using light microscopy and SEM.

The dissolving MAPs incorporating amodiaquine or artesunate were designed with a two-layer structure and fabricated using a double-casting technique. As shown in Fig. 3, formulations F1 (pure drug) and F2 (pure drug), which lacked Tween® 80 as a surfactant, exhibited structural defects. This suggests that the absence of a surfactant led to poor drug dispersion within the needle tips during the first-layer casting, particularly in the case of amodiaquine, resulting in an inhomogeneous aqueous mixture [39]. Consequently, this inconsistency likely contributed to needle fragility and breakage upon removal from the moulds. Notably, F1, which contained a drug-to-polymer (PVA and PVP) ratio of 1:2, appeared to create an overly dense polymeric network, making the microneedles brittle and prone to cracking [43]. In contrast, F2, F3, and F4, formulated with drug-to-polymer ratios of 1:1, 1:1, and 2:1, respectively, produced dissolving MAPs with well-defined microprojections on a smooth and stable baseplate. These formulations showed no visible air bubbles or irregular needle formations, indicating uniformity and structural integrity. This highlights the necessity of balancing drug and polymer concentrations to maintain mechanical stability, enhance drug loading, and ensure effective transdermal drug delivery. Therefore, for amodiaquine, only F2, F3, and F4 were selected for further evaluation and characterisation.

**Fig. 4:**
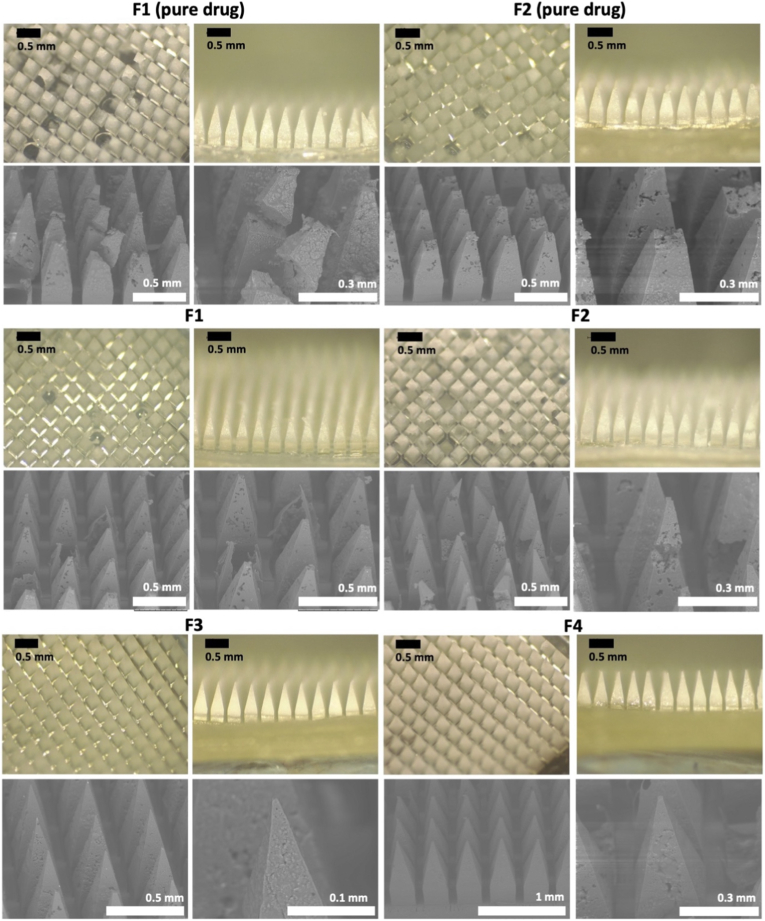
Optical characterisation of artesunate-loaded MAPs using light microscopy and SEM.

A similar pattern was observed for artesunate-loaded dissolving MAPs, where formulations lacking Tween® 80 (F1 and F2 for pure drug) failed to produce intact MAPs (Fig. 4). Given that artesunate has lower solubility than amodiaquine in deionised water (0.4 mg/mL *vs.* 4.8 mg/mL), this limited solubility likely led to drug aggregation or crystallisation within the microneedle matrix. This inhomogeneous drug distribution weakened the polymer structure, making the microneedles prone to fractures or breakage upon removal from the moulds [44]. These results highlight the critical role of solubility in microneedle formulations, influencing drug dispersion and polymer integrity. Poor solubility can cause defects due to phase separation, or weak polymer networks, whereas improving solubility (e.g., through surfactants) promotes uniform, mechanically stable microneedles for transdermal drug delivery. Furthermore, formulations with high polymer content (PVA and PVP K17) resulted in brittle microneedles, as observed in F1 and F2, despite the inclusion of Tween® 80. The dense polymeric network in these formulations likely increased brittleness and susceptibility to cracking. Additionally, a higher polymer concentration increases viscosity, which may hinder the complete filling of microneedle mould cavities, leading to defective needle tips upon demoulding. During the drying process, polymers tend to contract, potentially causing microneedle shrinkage and deformation. High PVA/PVP content further intensifies shrinkage, resulting in distorted microneedle shapes, such as curved, collapsed, or uneven tips. Therefore, only F3 and F3 for artesunate that given well-defined microprojections on a smooth baseplate. Accordingly, based on the morphology and appearance of the dissolving MAPs, only F3 and F4 were selected for further evaluation and characterisation in the case of artesunate.

### 3.3 Drug loading quantification

**Fig. 5:**
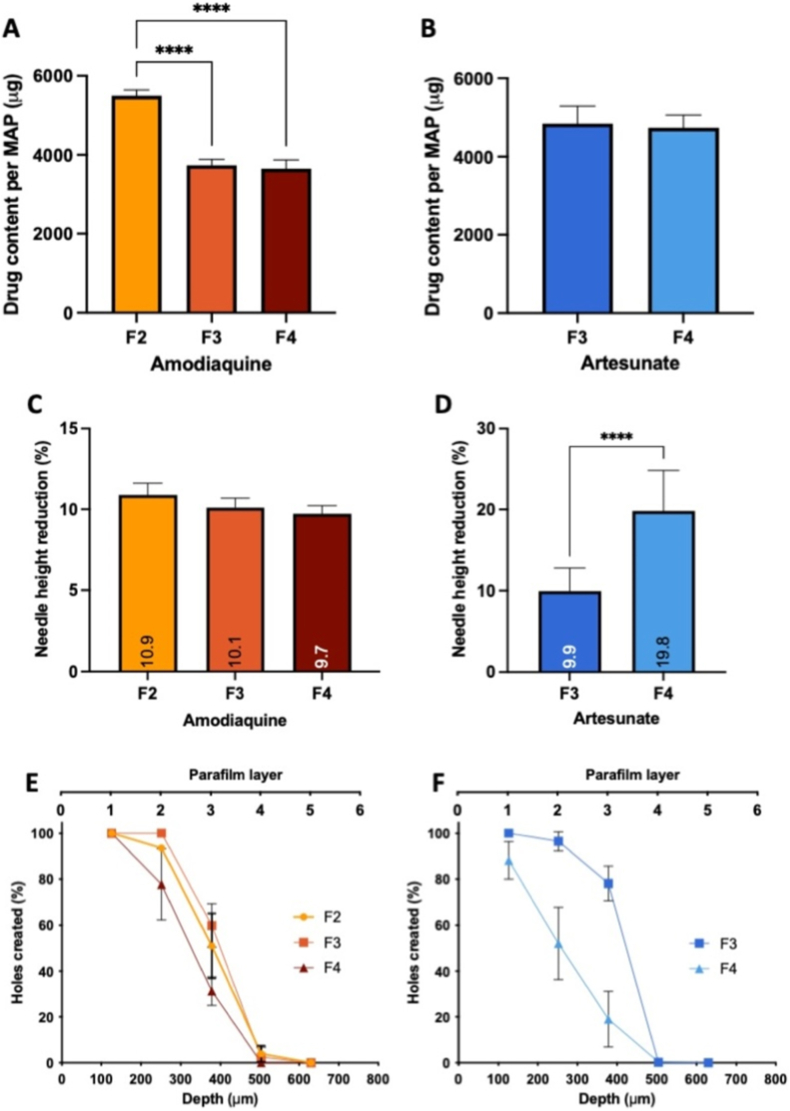
Comparison of drug content per MAP for (A) amodiaquine and (B) artesunate (means + SD, n ≥ 3). Percentage of needle height reduction after compression (32 N for 30 s) against a flat surface for (C) amodiaquine and (D) artesunate (means +SD, n = 30). Insertion capacity of dissolving MAPs in Parafilm® layers for (E) amodiaquine and (F) artesunate, measured by the number of holes created per layer (means ± SD, n = 3).

The dissolving MAPs with acceptable morphology were further evaluated for drug content. As depicted in Fig. 5A, amodiaquine loading in formulation F2 was significantly higher than in F3 and F4 (*p* < 0.05). This may be attributed to the higher solid content of F2 (56.1 % w/w, compared to 28.3 % w/w in F3 and 48.2 % w/w in F4). A higher solid content can promote more effective concentration of the drug-polymer blend into the needle tips during centrifugation. Specifically, a MAP patch measuring 0.36 cm^2^ and containing 256 needles exhibited amodiaquine loadings of 5.5 ± 0.1 mg, 3.7 ± 0.1 mg, and 3.6 ± 0.2 mg for F2, F3, and F4, respectively.

For artesunate, as presented in Fig. 5B, no significant difference in drug loading was observed between F3 and F4 (*p* > 0.05), with approximately 4.8 ± 0.4 mg and 4.7 ± 0.3 mg per MAP patch, respectively. Despite F4 having a higher drug concentration in the formulation, the limited solubility of artesunate may result in only a small portion dissolving within the polymer matrix, while the remainder may precipitate or aggregate, even with the inclusion of surfactant. This observation aligns with previous findings where the incorporation of surfactant did not significantly impact the loading of hydrophobic drugs, such as itraconazole, into MAPs [39].

### 3.4 Mechanical properties evaluation

The mechanical properties of dissolving MAPs was evaluated by measuring needle height reduction under compression to determine formulations with adequate structural integrity. As illustrated in Fig. 5C, amodiaquine-loaded MAPs (F2, F3, and F4) exhibited no significant differences in height reduction (*p* > 0.05). Conversely, artesunate-loaded MAPs displayed notable variability, with Fig. 5D indicating that the formulation with the highest drug loading (F4) was significantly more brittle than F3 (*p* < 0.05). This increased fragility may be attributed to the hydrophobic nature of artesunate, which has a lower solubility in Tween® 80 solution (∼1.3 mg/mL) compared to amodiaquine (∼7.9 mg/mL). Although Tween® 80 enhances drug solubility and reduces surface tension, the 0.5 % w/v concentration used may have been insufficient to fully dissolve artesunate, leading to partial drug aggregation within the microneedle matrix. Such aggregation can create inconsistencies in drug distribution, forming weak points that compromise mechanical integrity under applied pressure [45,46]. Furthermore, poorly soluble drugs tend to crystallise within the polymer matrix, generating rigid domains that disrupt polymer cohesion, thereby increasing susceptibility to fracture [47,48]. Consequently, higher drug loading in artesunate formulations resulted in greater needle height reduction, indicating diminished mechanical resilience. Based on these findings, F2, F3, and F4 of amodiaquine-loaded MAPs and F3 of artesunate-loaded MAPs exhibited a height reduction of less than 10 %, which aligns with established benchmarks for acceptable MAP mechanical performance [[49], [50], [51]]. This suggests that these formulations possess sufficient mechanical strength to endure compression forces during skin insertion.

### 3.5 Insertion capability assessment

To assess the insertion capability of MAPs, both Parafilm® and excised full-thickness neonatal porcine skin were used as skin simulants. The Parafilm® model consisted of an eight-layer stack with a total thickness of 1 mm, where each layer measured 125 μm. The percentage of holes created in the Parafilm® layers was quantified to determine the insertion efficiency of the MAPs. As depicted in Fig. 5E and F, the insertion profiles of amodiaquine-loaded and artesunate-loaded MAPs were evaluated, respectively.

For amodiaquine-loaded MAPs, all formulations achieved 100 % insertion efficiency into the first layer of Parafilm®, with no observable variations. Additionally, all formulations successfully penetrated down to the third layer of Parafilm®. Although no significant differences were observed in the insertion profiles (*p* > 0.05), F3 exhibited superior penetration, achieving 100 % insertion into the second layer (250 μm), whereas F2 and F4 reached 93 % and 77 %, respectively.

For artesunate-loaded MAPs, a significant difference was noted between F3 and F4 (*p* < 0.05), with F3 demonstrating superior insertion efficiency. Specifically, 78 % of holes were created in the third layer of Parafilm® for F3, while F4 exhibited lower efficiency, with only 88 % in the first layer, 52 % in the second layer, and 19 % in the third layer. This finding aligns with the mechanical resistance data, where F4 demonstrated greater needle height reduction, indicating increased brittleness due to high drug loading of the poorly soluble artesunate in the needle tips. This brittleness likely compromised the insertion capability of F4.

**Fig. 6:**
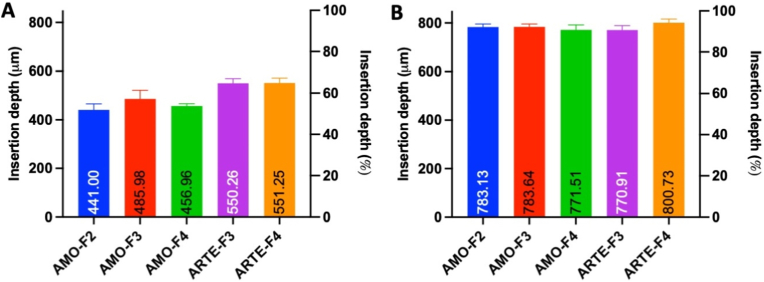
Insertion depth of amodiaquine (AMO)-loaded and artesunate (ARTE)-loaded MAPs into (A) Parafilm® and (B) full-thickness porcine skin, measured by OCT (means + SD, n = 20). (For interpretation of the references to colour in this figure legend, the reader is referred to the Web version of this article.)

Insertion of MAPs into both Parafilm® and excised neonatal porcine skin was visualised using OCT. Insertion depth was assessed by measuring the gap between the baseplate and the surface of Parafilm® or porcine skin, with the uninserted portion of the microneedles quantified. The data, presented in Fig. 6A and B, revealed insertion depths of approximately 440–550 μm in Parafilm® and 780–800 μm in excised neonatal porcine skin, corresponding to approximately 60 % and 95 % of the needles being inserted, respectively. Notably, MAPs penetrated deeper into excised neonatal porcine skin compared to Parafilm® across all formulations. This discrepancy may be attributed to the material properties of Parafilm®, which is derived from paraffin wax and does not fully replicate the mechanical characteristics of human skin [28,29]. Moreover, MAPs are composed of hydrophilic polymers that encapsulate the drug, and the presence of interstitial fluid in neonatal porcine skin may facilitate hydration, rendering the structure more flexible and enhancing insertion compared to the dry, moisture-deficient Parafilm® [39]. Despite these differences, Parafilm® remains a validated model for MAP insertion studies and serves as a reliable tool for comparative formulation assessments [29]. The findings confirm that all MAP formulations successfully inserted into skin simulants. Considering the overall evaluation results, F2 for amodiaquine and F3 for artesunate were selected for further investigation.

### 3.6 *In situ* skin dissolution study

**Fig. 7:**
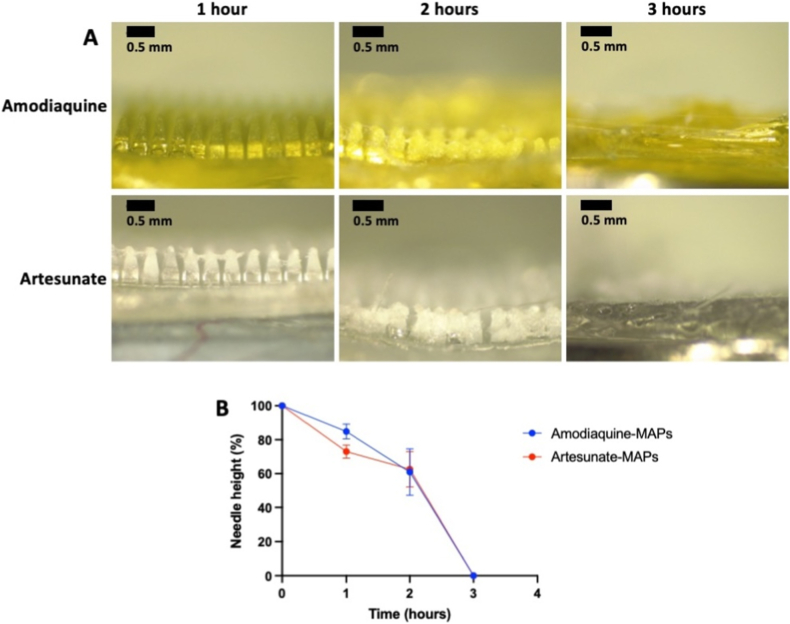
(A) Representative images of *in situ* skin dissolution of amodiaquine-loaded MAPs and artesunate-loaded MAPs at 1 h, 2 h, and 3 h. (B) Dissolution rate of the microneedles, expressed as the percentage reduction in needle height over time (means ± SD, n ≥ 10).

The selected formulations of amodiaquine- and artesunate-loaded MAPs were evaluated for *in situ* skin dissolution to determine the time required for complete dissolution upon application (Fig. 7A). As depicted in Fig. 7B, no significant differences were observed in the dissolution kinetics between the two formulations (*p* > 0.05). After 1 h of skin application, needle height was reduced to approximately 85 % for amodiaquine and 73 % for artesunate. By 2 h, needle height reduction reached around 61 % and 63 % for amodiaquine- and artesunate-loaded MAPs, respectively. Complete dissolution of the needle layer was observed within 3 h. Notably, if the patch remained on the skin beyond this period, full dissolution of both the microneedle layer and the polymeric baseplate occurred, forming an occlusive, adhesive polymeric gel on the skin surface.

### 3.7 Dermatokinetic study

**Fig. 8:**
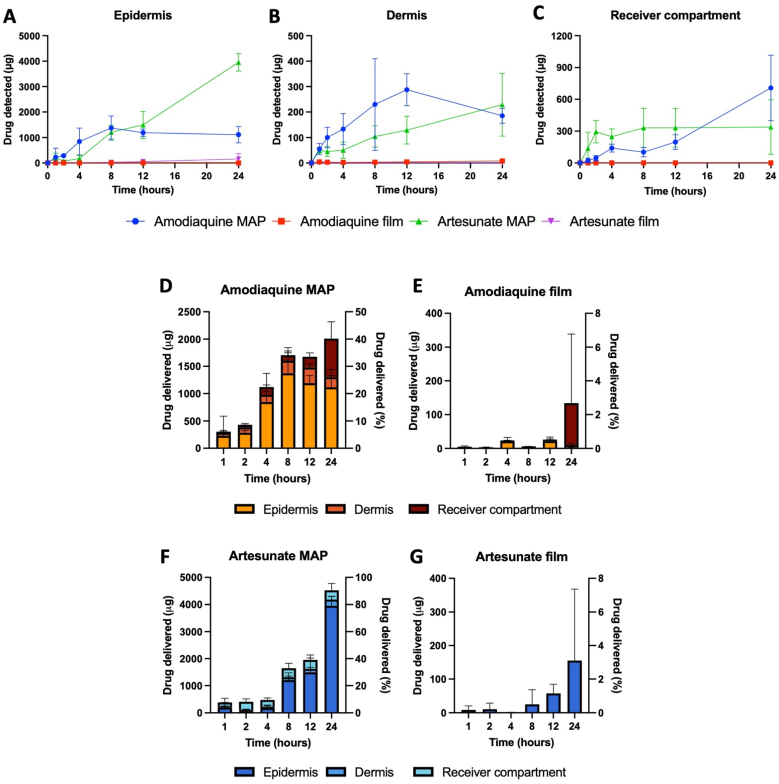
*Ex vivo* dermatokinetic evaluation of dissolving MAPs for transdermal delivery across full-thickness neonatal porcine skin. (A–B) Drug deposition profiles of amodiaquine and artesunate from MAPs and control films in the epidermis and dermis, respectively (means ± SD, n ≥ 3). (C) Permeation profiles of amodiaquine and artesunate from MAPs and films over time (means ± SD, n ≥ 3). (D–G) Cumulative drug amounts (amodiaquine and artesunate) deposited in the epidermis, dermis, and permeated through the skin from MAPs and films (means +SD, n ≥ 3). The delivered drug amount (μg) is plotted on the primary y-axis, while the percentage delivery efficiency is represented on the secondary y-axis (means + SD, n ≥ 3).

The permeation and deposition profiles of amodiaquine and artesunate across different skin layers were evaluated at predefined time points. As a control, thin films containing either drug, but no microneedles were used. The drug deposition in the epidermis is depicted in Fig. 8A. Minimal drug accumulation was observed when films were used, with approximately 3.1 ± 2.7 μg of amodiaquine and 155.1 ± 212.7 μg of artesunate detected over 24 h. In contrast, MAP-loaded amodiaquine demonstrated sustained and steady drug deposition, reaching 1116.0 ± 324.1 μg by 24 h. For artesunate-MAPs, a linear increase in drug deposition was observed from 4 to 24 h, with a total of 3954.7 ± 342.1 μg detected in the epidermis.

In the dermis, no drug was detected from the film-based samples for either drug. While no statistically significant difference was noted in drug deposition between amodiaquine and artesunate MAPs (*p* > 0.05), amodiaquine levels in the dermis declined from 12 to 24 h, whereas artesunate continued to accumulate. At 24 h, 185.4 ± 29.1 μg of amodiaquine and 228.7 ± 123.6 μg of artesunate were detected in the dermis. Drug permeation across the full-thickness skin was sustained for artesunate, with a total permeation of approximately 350 μg. In contrast, while amodiaquine-MAP permeation was initially lower, it increased steadily from 8 to 24 h, with a total of approximately 710 μg permeated.

As shown in Fig. 8D–G, the total amount of amodiaquine delivered over 24 h was approximately 2000 μg, with a delivery efficiency of 40 %. In contrast, films, which rely solely on passive diffusion, delivered only approximately 170 μg (2.4 %). Interestingly, artesunate MAPs achieved a significantly higher total delivery of approximately 4500 μg (90 %), compared to 180 μg (3.1 %) from films. The greater delivery of artesunate *via* MAPs compared to amodiaquine can be attributed to differences in their BCS. Artesunate, classified as a BCS Class II drug (high permeability, low solubility), benefits from the solubilising effects of Tween® 80, enhancing its dissolution kinetics and overall transdermal delivery. Amodiaquine, a BCS Class III drug (high solubility, low permeability), faces greater challenges in crossing the skin barrier, leading to lower overall absorption [21,22]. These findings highlight the superior drug delivery potential of dissolving MAPs compared to passive diffusion-based films, emphasising their promise in improving transdermal administration of amodiaquine and artesunate for malaria treatment.

### 3.8 Biocompatibility study

**Fig. 9:**
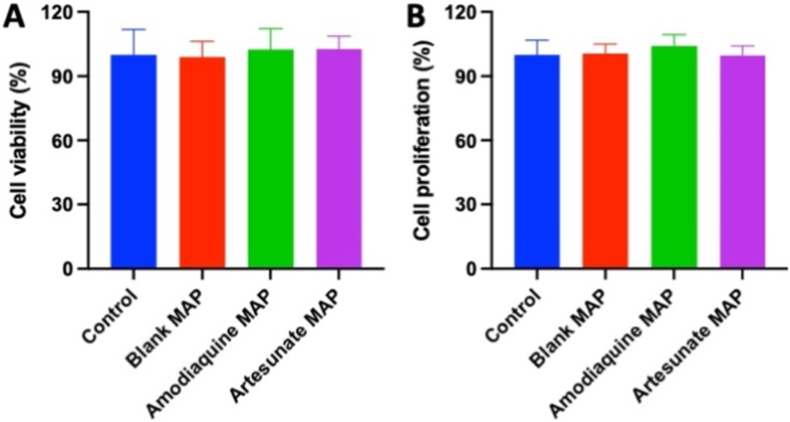
(A) Percentage of viable cells after 72 h of culture with blank MAP, amodiaquine MAP and artesunate MAP in the MTT assay (means + SD, n = 6). (B) Total DNA content of cells for control, blank MAP, amodiaquine MAP and artesunate MAP after 72 h of culture in the PicoGreen assay (means + SD, n = 6).

4. In this study, an MTT assay was employed to evaluate the cytotoxic effects of the formulations (blank MAP, amodiaquine MAP and artesunate MAP) on human dermal cells. As illustrated in Fig. 9A, the MTT results revealed cell viability percentages of 102.5 ± 6.0 % for amodiaquine MAP and 102.6 ± 9.6 % for artesunate MAP after 72 h. Statistical analysis indicated no significant differences between the control and any of the formulations (*p* > 0.05), and the cytotoxicity level was classified as 0 according to ISO 10993-5, as previously described [52,53]. Furthermore, a PicoGreen assay was performed to evaluate the effect of the formulations on healthy cell proliferation over a 72-h period. The results indicated that exposure to blank MAP, amodiaquine MAP, and artesunate MAP had no adverse effect on cell proliferation, with proliferation rates of 100.7 ± 4.3 %, 104.1 ± 5.3 %, and 99.7 ± 4.3 % for the respective formulations (Fig. 9B). This observation, combined with the results from Fig. 9A and B, confirmed that exposure to the formulations did not compromise cell viability or plasma membrane integrity. These findings suggest a high likelihood of safe use for all formulations without causing skin damage. Collectively, these results demonstrate that the tested formulations lack cytotoxic properties toward keratinocytes in human skin, indicating that amodiaquine MAP and artesunate MAP are likely safe for human application.

### 3.9 *In vivo* pharmacokinetic study

**Fig. 10:**
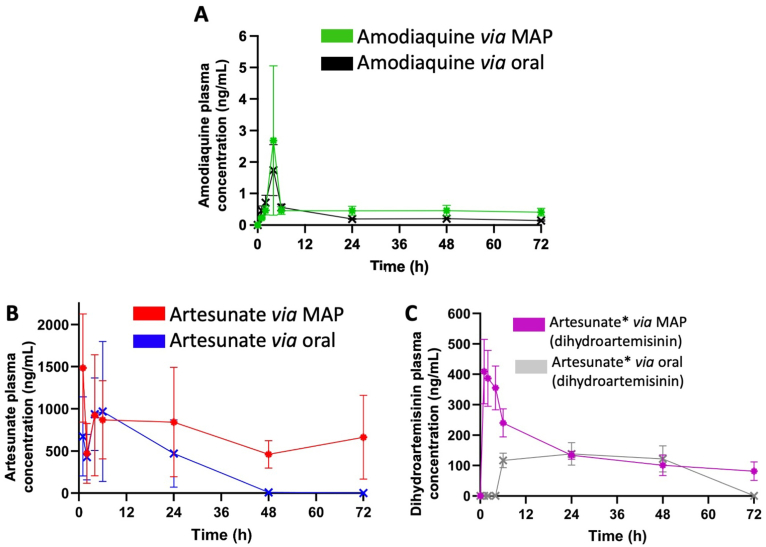
Pharmacokinetic profiles of (A) amodiaquine, (B) artesunate, and (C) dihydroartemisinin following administration *via* MAPs and oral suspensions, with measurements recorded over a 72-h period. Single doses of both MAPs and oral formulations were administered on Day 1 of treatment (means ± SEM, *n* = 6). The total dose administered per animal *via* MAPs was 22 mg for amodiaquine and 19.2 mg for artesunate. For the oral control group, rats received a single dose of amodiaquine (10 mg/kg) and artesunate (4 mg/kg) by oral gavage.

The pharmacokinetic profiles of amodiaquine and artesunate were assessed individually. Fig. 10 and Table 3 illustrate the rat pharmacokinetic parameters of these drugs delivered *via* dissolving MAP formulations, compared with oral administration, the standard pharmaceutical approach for the treatment and prevention of malaria caused by *P. falciparum*. This drug combination is endorsed by the WHO as the gold standard for *P. falciparum* treatment, especially in paediatric cases [5]. However, oral artesunate has limited bioavailability (approximately 30 %) and a short half-life of around 1 h in humans [54,55].

**Table 3:** Pharmacokinetic parameters of amodiaquine, artesunate, and dihydroartemisinin following a single dose of either oral suspension or MAP application. Oral administration included amodiaquine (3 mg/rat, n = 6) and artesunate (1.2 mg/rat, n = 6). MAP administration included amodiaquine MAPs (22 mg/rat, n = 6) and artesunate MAPs (19.2 mg/rat, n = 6). Data presented in means ± SEM, *n* = 6.

Drugs/metabolite∗	Amodiaquine		Artesunate		Dihydroartemisinin∗	
PK parameters/formulation	Oral amodiaquine	MAP amodiaquine	Oral artesunate	MAP artesunate	Oral artesunate	MAP artesunate
AUC (ng/ml∗h)	21.37 ± 0.91	36.26 ± 8.85	22844.75 ± 4840.58	40779.24 ± 8436.53	4264.25 ± 1333.46	7730.24 ± 2364.20
C_max_ (ng/ml	2.13 ± 0.92	3.00 ± 2.31	1421.93 ± 209.61	1796.01 ± 154.49	172.97 ± 12.98	559.03 ± 116.69
T_max_ (h)	4.00 ± 0.63	22.00 ± 12.42	4.00 ± 1.00	7.75 ± 3.60	16.80 ± 4.41	1.67 ± 0.49
t_1/2_ (h)	40.75 ± 7.44	57.72 ± 19.16	6.37 ± 0.75	54.05 ± 17.32	24.85 ± 1.22	22.48 ± 5.23
MRT (h)	50.80 ± 7.62	87.95 ± 28.16	13.27 ± 1.75	84.99 ± 28.01	28.85 ± 1.84	31.54 ± 7.81

In this study, a significantly longer half-life of artesunate was observed following MAP delivery compared to oral administration (54.05 ± 17.32 h *vs.* 6.37 ± 0.76 h). Interestingly, artesunate *via* oral administration was still able to maintain detectable plasma levels of artesunate and its metabolite for up to 13.27 h and 28.85 h, respectively, likely due to its suspension in 1 % carboxymethylcellulose sodium (NaCMC), a known release modifier. Previous studies have demonstrated that CMC improves the stability and bioavailability of artemisinin derivatives [56]. For the MAP group, artesunate and its metabolite maintained detectable plasma levels for up to 84.99 h and31.54 h, respectively.

In the oral group, artesunate reached a C_max_ of 1421.93 ± 209.62 ng/mL at 4 h, with a half-life of 6.37 h. Its active metabolite, dihydroartemisinin (DHA), achieved a C_max_ of 172.97 ± 12.98 ng/mL at 16.80 h, with a half-life of 24.85 h. In contrast, MAP administration exhibited higher plasma concentrations and prolonged exposure: artesunate delivered *via* MAPs reached a C_max_ of 1796.01 ± 154.49 ng/mL at 7.75 h, with a markedly extended half-life of 54.05 h, an 8.49-fold increase relative to oral dosing. Dihydroartemisinin (DHA) levels following MAP application peaked earlier at 1.67 h, with a significantly higher C_max_ of 559.03 ± 116.69 ng/mL, compared to the oral route (C_max_ 172.97 ± 12.98 ng/mL at 16.80 h). These findings suggest that artesunate-loaded MAPs offer sustained systemic availability, potentially improving parasite clearance. This sustained delivery of artesunate and DHA may provide a therapeutic advantage in addressing artemisinin resistance, which often necessitates modified dosing regimens and combination strategies with partner drugs [57].

To prolong antimalarial efficacy, artesunate is typically paired with longer-acting agents such as amodiaquine, which also provides liver-stage protection. Recent clinical studies report oral amodiaquine half-lives between 32 and 72 h and C_max_ values around 18.8 ng/mL [58]. In our study, oral amodiaquine exhibited a half-life of 40.75 ± 7.44 h, a C_max_ of 2.13 ± 0.92 ng/mL, and a T_max_ of 4 h. In contrast, MAP delivery extended the half-life to 57.72 ± 19.16 h, with a higher C_max_ of 3.00 ± 2.31 ng/mL, and nearly doubled the mean residence time (MRT) compared to oral dosing (87.95 h *vs.* 50.80 h).

This parameter is critical, as the prolonged presence of amodiaquine in the bloodstream is essential for eliminating residual parasites and preventing the emergence of drug resistance. Artemisinin resistance has already become a significant concern in numerous malaria-endemic regions, particularly those affected by *Plasmodium falciparum*. Resistance to artemisinin, one of the most widely used antimalarial agents, has been reported in Southeast Asia and, more recently, in sub-Saharan Africa, posing a serious threat to the effectiveness of current treatment regimens [57,59]. This resistance often manifests as delayed parasite clearance from the bloodstream, potentially leading to treatment failure and the development of multidrug-resistant strains. Contributing factors include improper drug use, subtherapeutic dosing, and the rapid metabolism of artemisinin derivatives, which limits their duration of action in the body [57].

To address this growing concern, novel drug delivery systems such as MAPs offer a promising alternative by enabling controlled, extended release of both amodiaquine and artesunate. MAPs are minimally invasive, easy to apply, and capable of delivering a sustained release of antimalarial drugs into the bloodstream. This slow-release mechanism maintains therapeutic drug concentrations over longer durations, potentially enhancing efficacy and reducing the risk of resistance by avoiding suboptimal plasma levels. Previous studies have shown that microneedle-based formulations can improve pharmacokinetics by prolonging the half-life and increasing drug residence time in the body, thereby lowering the likelihood of parasite recrudescence and resistance development [18,19]. By providing more consistent drug exposure, MAPs could help mitigate artemisinin resistance and improve malaria treatment outcomes.

In confirmed cases of resistance, such as those involving Plasmodium strains with K13 mutations, the WHO recommends shifting the partner drug from lumefantrine to amodiaquine. However, the most promising strategy currently under evaluation is the 3-day triple artemisinin-based combination therapy (TACT), which combines one artemisinin derivative with two partner drugs, such as lumefantrine and amodiaquine [57].

### 3.10 Histopathology

**Fig. 11:**
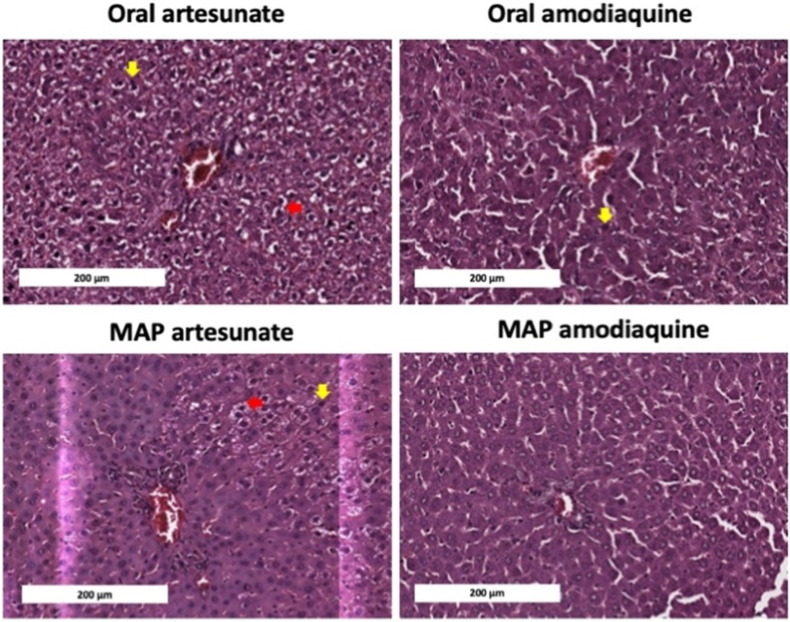
Representative histological images of rat liver sections from different treatment groups: oral artesunate, oral amodiaquine, MAP artesunate, and MAP amodiaquine. Images were captured at 40 × magnification with a scale bar of 200 μm. Red arrows indicate liver vacuolisation, while yellow arrows highlight areas of liver necrosis. (For interpretation of the references to colour in this figure legend, the reader is referred to the Web version of this article.)

This study, as presented in Fig. 11, revealed that oral administration of artesunate induced hepatocyte cell death, as indicated by the red arrows, and hepatocyte vacuolisation, as shown by the yellow arrows. Artesunate likely triggered hepatocyte cell death, specifically ferroptosis, through a reduction in glutathione (GSH) levels and increased oxidative stress, mediated by the activation of the transcriptional factor ATF3 [60]. In contrast, rats treated with MAP exhibited significantly less hepatocyte cell death and liver vacuolisation. The use of the MAP not only enhanced the efficacy of artesunate, but also minimised its side effects. Additionally, this study investigated the effects of amodiaquine on liver toxicity. Liver vacuolisation is known to precede liver injury. Here, we demonstrated that oral administration of amodiaquine also induced liver vacuolisation (yellow arrows), followed by hepatocyte cell death (red arrows), through the generation of oxidative stress [61]. Once again, the use of the MAP technology mitigated the side effects of oral amodiaquine and artesunate, as evidenced by reduced cell death and minimal vacuolisation.

### 3.11 *In vivo* antimalarial activity in Plasmodium berghei-infected mice

**Fig. 12:**
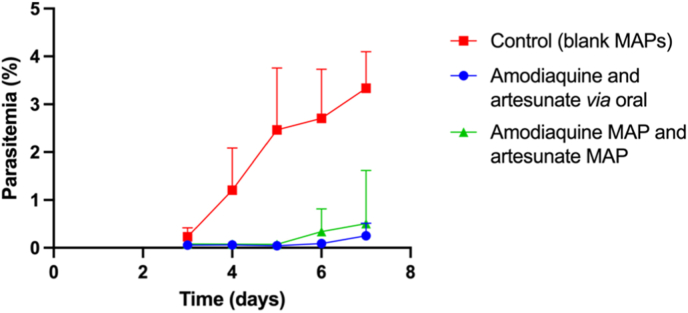
Parasitemia progression over seven days post-infection in mice infected with *Plasmodium berghei*. The control group received a blank MAP (drug-free), while treatment groups were administered amodiaquine and artesunate either orally or *via* MAPs (means + SD, n = 7).

After evaluating the *in vitro* and pharmacokinetic profiles of dissolving MAPs loaded with either amodiaquine or artesunate, we proceeded to assess their efficacy in eliminating *Plasmodium berghei* in infected mice (Fig. 12). This study aimed to determine whether incorporating these drugs into the polymeric tips of MAPs affected their antimalarial activity when compared to conventional oral administration. The results demonstrated that both amodiaquine and artesunate retained their efficacy after being formulated into MAPs. The combination of amodiaquine- and artesunate-loaded MAPs achieved a 99.5 % reduction in parasitaemia, with no statistically significant difference in parasite clearance compared to the group receiving oral drug administration (*p* > 0.05). However, a significant difference was observed between the drug-loaded MAP group and the blank MAP group (*p* < 0.05), confirming that active drug delivery was essential for effective treatment.

In this study, single-drug MAPs (containing only amodiaquine or artesunate) were not tested, as effective malaria treatment, particularly for *P. falciparum*, the most lethal malaria-causing parasite, requires combination therapy [62]. Artesunate is a fast-acting antimalarial that rapidly reduces parasite levels in the bloodstream, but it has a short half-life and is quickly eliminated from the body [63]. In contrast, amodiaquine, through its active metabolite desethylamodiaquine, acts more slowly and remains in the system for a longer period, ensuring clearance of residual parasites and reducing the risk of reinfection [64].

When used together, artesunate provides an immediate reduction in parasitaemia, while amodiaquine sustains therapeutic levels, thereby minimising the potential for drug resistance. Notably, the MAPs developed in this study demonstrated the ability to maintain therapeutic drug effect in the bloodstream for up to seven days in the mice model, enabling effective parasite elimination. This extended release also contributed to reduced hepatic toxicity, as confirmed by histological analysis (Fig. 11). These findings support the potential of MAPs as a promising alternative strategy for malaria treatment, combining sustained drug release, efficacy, and improved patient adherence.

Building on this proof-of-concept study, future work will focus on optimising the MAP platform to enhance delivery efficiency and clinical translatability. Key areas include refining drug loading strategies to reduce the required patch size while maintaining therapeutic plasma levels, and adjusting formulation parameters to achieve better control over release kinetics. Furthermore, evaluating the MAP system in larger animal models and under repeat-dose regimens will be critical for supporting clinical translation. From a programmatic standpoint, integration of MAPs into existing malaria treatment frameworks could provide a simplified, adherence-friendly alternative to oral therapies, particularly for paediatric and remote populations. Ultimately, this technology has the potential to be adapted for other antimalarial drug combinations or for TACTs, offering a robust platform to help address growing concerns around drug resistance and treatment failure.

## 4 Conclusion

This study successfully developed and characterised dissolving MAPs individually loaded with amodiaquine and artesunate in the needle tips, incorporating the surfactant Tween® 80 to enhance drug solubility within the aqueous formulation. This strategy was designed to provide an alternative to oral regimens that require multiple doses over several days, which can reduce adherence, compromise parasite clearance, and contribute to the emergence of drug resistance. The inclusion of Tween® 80 improved solubility, skin permeability, and the formation of well-defined microprojections for both drugs. The drug content per 0.36 cm^2^ MAP ranged from 3.6 to 5.5 mg for amodiaquine and 4.7–4.8 mg for artesunate. Mechanical testing showed that all amodiaquine MAP formulations and the F3 artesunate MAP exhibited less than 10 % height reduction, consistent with acceptable standards for MAP mechanical integrity. All formulations successfully inserted into skin simulants, and *in situ* dissolution studies confirmed complete needle dissolution within 3 h. Dermatokinetic analysis demonstrated that approximately 40 % of amodiaquine and 90 % of artesunate permeated and deposited into the skin within 24 h, indicating the potential for sustained intradermal drug release. *In vivo* pharmacokinetic studies in rats further confirmed that a single 24-h MAP application maintained plasma concentrations for over three days. Moreover, efficacy studies in *Plasmodium berghei*-infected mice showed that artesunate- and amodiaquine-loaded MAPs reduced parasitaemia by up to 99.5 %, comparable to oral administration. Overall, these findings demonstrate that dissolving MAPs represent a promising, minimally invasive, and needle-free drug delivery system for malaria treatment. This approach could significantly improve patient adherence and drug accessibility, particularly in remote or resource-limited settings where oral or injectable therapies present logistical challenges.

## CRediT authorship contribution statement

**Qonita Kurnia Anjani:** Writing – review & editing, Writing – original draft, Visualization, Validation, Resources, Project administration, Methodology, Investigation, Formal analysis, Data curation, Conceptualization. **Fabiana Volpe-Zanuto:** Writing – review & editing, Writing – original draft, Validation, Resources, Methodology, Formal analysis, Data curation, Conceptualization. **Andang Miatmoko:** Resources, Project administration, Methodology. **Natalia Moreno-Castellanos:** Writing – original draft, Methodology, Formal analysis, Data curation. **Janaina Tenorio Novais:** Methodology, Investigation, Formal analysis, Data curation. **Xiomara A. Gaitán:** Methodology, Investigation, Formal analysis, Data curation. **Berlian Sarasitha Hariawan:** Methodology, Investigation, Formal analysis, Data curation. **Devy Maulidya Cahyani:** Methodology, Investigation, Formal analysis, Data curation. **Rifda Tarimi Octavia:** Methodology, Investigation, Formal analysis, Data curation. **Ahmad Shahrul Mubarok:** Methodology, Investigation, Formal analysis, Data curation. **Wiwit Ananda Wahyu Setyaningsih:** Writing – original draft, Methodology, Investigation, Formal analysis, Data curation. **Callum Johnston:** Writing – review & editing. **Daniel Y. Bargieri:** Writing – review & editing, Supervision, Resources, Project administration, Funding acquisition, Conceptualization. **Ryan F. Donnelly:** Writing – review & editing, Supervision, Resources, Project administration, Funding acquisition, Conceptualization.

## Disclosure

The term “microneedles” encompasses several distinct technologies. Ryan F. Donnelly, through his university, has licensed patents on specific microneedle platforms to individual companies and has provided consultancy advice to companies developing microneedle-based products on a case-by-case basis. The microneedle systems described in this manuscript are not covered by any patent (out-licensed or otherwise) held by Ryan F. Donnelly, nor does he provide consultancy advice to any company developing microneedle technologies for delivery of amodiaquine, artesunate or any other antimalarial drug. Accordingly, Ryan F. Donnelly declares no conflict of interest in relation to this work. Also, the other authors declare no conflict of interest.

## Declaration of competing interest

The authors declare that they have no known competing financial interests or personal relationships that could have appeared to influence the work reported in this paper.

## Data Availability

All data created during this research are openly available at http://doi.org/10.17034/2dd2723f-ccd5-4e56-bc2d-c405323a25df.
